# Micro-Environmental Signature of The Interactions between Druggable Target Protein, Dipeptidyl Peptidase-IV, and Anti-Diabetic Drugs

**DOI:** 10.22074/cellj.2016.4865

**Published:** 2016-12-21

**Authors:** Chiranjib Chakraborty, Bidyut Mallick, Ashish Ranjan Sharma, Garima Sharma, Supriya Jagga, C George Priya Doss, Ju-Suk Nam, Sang-Soo Lee

**Affiliations:** 1 Institute for Skeletal Aging and Orthopedic Surgery, Hallym University-Chuncheon Sacred Heart Hospital, Chuncheon, Korea; 2 Department of Bio-Informatics, School of Computer and Information Sciences, Galgotias University, Greater Noida, India; 3 Departments of Physics, Galgotias College of Engineering and Technology, Greater Noida, India; 4 Department of Integrative Biology, VIT University, Vellore Tamil Nadu, India

**Keywords:** Dipeptidyl Peptidase-IV (DPP-4), Saxagliptin, Linagliptin, Vildagliptin

## Abstract

**Objective:**

Druggability of a target protein depends on the interacting micro-environment between the target protein and drugs. Therefore, a precise knowledge of the interacting micro-environment between the target protein and drugs is requisite for drug discovery process. To understand such micro-environment, we performed in silico interaction analysis between a human target protein, Dipeptidyl Peptidase-IV (DPP-4), and three anti-diabetic drugs (saxagliptin, linagliptin and vildagliptin).

**Materials and Methods:**

During the theoretical and bioinformatics analysis of micro-environmental properties, we performed drug-likeness study, protein active site predictions, docking analysis and residual interactions with the protein-drug interface. Micro-environmental landscape properties were evaluated through various parameters such as binding energy, intermolecular energy, electrostatic energy, van der Waals’+H-bond+desolvo energy (E_VHD_) and ligand efficiency (LE) using different in silico methods. For this study, we have used several servers and software, such as Molsoft prediction server, CASTp server, AutoDock software and LIGPLOT server.

**Results:**

Through micro-environmental study, highest log P value was observed for linagliptin (1.07). Lowest binding energy was also observed for linagliptin with DPP-4 in the binding plot. We also identified the number of H-bonds and residues involved in the hydrophobic interactions between the DPP-4 and the anti-diabetic drugs. During interaction, two H-bonds and nine residues, two H-bonds and eleven residues as well as four H-bonds and nine residues were found between the saxagliptin, linagliptin as well as vildagliptin cases and DPP-4, respectively.

**Conclusion:**

Our in silico data obtained for drug-target interactions and micro-environmental signature demonstrates linagliptin as the most stable interacting drug among the tested anti-diabetic medicines.

## Introduction

Productivity of the pharmaceutical industry revolves around the discovery of new pharmaceutical entities (NMEs). It has been observed that Food and Drug Administration (FDA), approved NMEs, are shrinking with the passage of time. Therefore, current rate of productivity of pharmaceutical industries is alarming and development of new NMEs is the contemporary call from the pharmaceutical industries (1). In depth understanding of drug target can help us to develop the quality of NMEs at a faster rate and thus may contribute to fulfill the increasing demand of new feasible NMEs for the pharmaceutical industry. Henceforth, several pharmaceutical companies are taking new initiatives in this direction. One such example is Bayer Health Care’s 'Grants4Targets' initiative, launched in year 2009. In this proposal, company campaigned to provide drug discovery knowledge and support academic world for assessment and validation of novel drug targets (2,3). 

Drug discovery through target evaluation and validation has already shown a pathway toward major successes. It has been observed that several drugs are developed after understanding that human target proteins are currently available in the market and numerous other drug targets are being identified in this prospective. Indeed, a number of current human drug targets interacting with small molecules, approximately 200 to 500 in terms of quantity, have been identified and confirmed from the literature (4). In the year 1996, Drews (5) was the first to analyze potential target proteins in humans as well as in pathogens, and reported about 483 target proteins. Thereafter, in 2002 Hopkins and Groom performed another analysis and identified 399 molecular targets from 130 protein families. This study described the molecular targets and their ligands having druglike properties (6). Consequently, Golden (7,8) projected that all of the approved drugs which is available in the market act through 273 proteins. In 2006, another group of researchers documented about 218 molecular targets for approved drugs (9). However, Zheng et al. (10,11) recorded 268 'successful' targets from the therapeutic targets database and Overington et al. (12) suggested a compromise number of 324 drug targets from all classes of approved drugs for only therapeutic purposes. The theory of druggable targets, projected by Hopkins and Groom (6), is crucial for drug discovery and is based on the 'rule-of-five' analysis of drug-likeness as proposed by Lipinski et al. (13). It has been revealed that approximately 60% of small molecule drug discovery projects were not successful, since the target was found to be non-druggable. 

Druggable targets and the targetability of drugs are the two most significant factors required to determine the efficacy of new small molecules (14,15). Thomson Reuters Life Science Consultancy (Pharma Consulting Services) has investigated unsuccessful phase II projects of drug discovery during 2008 to 2010 and noted that about 51% of failures occurred due to insufficient efficacy of newly discovered drugs (16). It means that, interaction efficacy between drug-like molecule and druggable target were not appropriate. It is well known that micro-environmental signature of the interactions between druggable target protein and the drug is the most crucial event for its medicinal activity. Therefore, a detailed understanding of the micro-environmental landscape interaction between druggable target proteins and drugs is a prerequisite for successful drug discovery. 

The interaction landscape of target protein and drug depends on peculiar micro-environmental factors such as binding energy, intermolecular energy, electrostatic energy, van der Waals’ interaction energy, Hydrogen (H)-bond, desolvo energy and ligand efficiency (17). One of the important factors during interaction is binding energy; it can help to understand the binding affinity between target and drug (18). Intermolecular force between two molecules is another micro-environmental factor. Leckband (19) described a vital role of intermolecular forces or energy during protein interaction with ligand complex. Through analysis of binding sites, researchers can illustrate binding affinity between any two molecules. It has been observed that highaffinity for drug and target binding results from the greater intermolecular force. On the other hand, low-affinity ligand binding involves less intermolecular force between drug and target (20). Furthermore, another imperative factor during interaction is electrostatic communication between cations and anions. It can be measured during functional analysis of biological molecules (21). It is well known that van der Waals’ interaction energy represent a sum up of the attractive forces or repulsive forces between the molecules. H-bonds are also a significant factor to understand the micro-environment interaction. Significance of H-bonds during target-drug interaction have already been described (22). Ligand efficiency, capacity of binding energy per atom unit, has lately emerged as a useful guide to lead selection in the drug discovery process (23). All of these factors have been described from time to time either as a single factor or in combination. However, how all of these factors optimize interactions between drug-target, especially in anti-diabetic drugs has not properly been analyzed. 

Throughout this decade, diabetes is a severe health crisis and the number of diabetes patients is growing worldwide at an alarming rate (24). Currently, in both types 1 and 2 diabetes, 366 million people are affected around the world (25) and it is predicted to increase up to 552 million by 2030. It has been noted that 90-95% of population, among the total diabetes, are suffering type 2 diabetes (T2D) (26). Current treatments for T2D include administration of several therapeutic agents as well as endeavoring to modify lifestyle. Among the various available line of treatment for T2D, "incretins" is one of the best available choices. Incretins are a class of gastrointestinal hormones that directly stimulates insulin secretion and decreases glucose level. This class of gastrointestinal hormones comprises of two hormones, including glucagon-like peptide-1 (GLP-1) and glucose-dependent insulinotropic polypeptide (GIP, also called gastric inhibitory polypeptide), with an anti-diabetic role for both of them. These hormones increase insulin secretion and helps in proliferation of pancreatic β-cell. But, dipeptidyl peptidase-IV (DPP-4) can degrade GLP-1 and GIP protein molecules, quickly (27). Hence, DPP-4 is a major drug target for treating T2D. Regarding that some anti-diabetic drugs (e.g. sitagliptin, vildagliptin, saxagliptin, linagliptin) act as an inhibitor of the DPP-4, they are preferred by the physician in the management of T2D (28). 

In this article, we tried to understand micro-environmental signature of interactions between druggable target, DPP-4, and three anti-diabetic drugs (i.e. saxagliptin, linagliptin and vildagliptin). For that, we firstly analyzed drug-likeness of our selected anti-diabetic drugs and the predictive active site on the targeted protein. For depiction of micro-environmental landscape during these interactions, we have evaluated different micro-environmental parameters such as binding energy, intermolecular energy, cumulative sum of electrostatic energy, van der Waals’+H-bonds+desolvo energy (E_VHD_) and ligand efficiency (LE). Finally, we analyzed the residual interactions at the protein-drug interface between DPP-4 and three anti-diabetic drugs.

## Materials and Methods

### Target protein and drugs section

In order to understand the interacting microenvironmental signature involved in drug-target binding, a theoretical and bioinformatics study was performed. For this purpose, we selected a target protein, human DPP-4, as diabetes drug target. DPP-4 format file (pdb id: 1j2e) was retrieved from Protein Data Bank (PDB, www.rcsb.org) for further analysis (29). 

Three existing anti-diabetic drugs and inhibitors of DPP-4 (saxagliptin, linagliptin and vildagliptin) were selected for this study. Drug information (including 3D or 2D structure, and canonical SMILES data) were obtained from drug bank database (30), as well as PubChem. 

### Drug-likeness analysis of the selected anti-diabetic drugs

Drug-likeness and molecular properties of three existing anti-diabetic drugs were calculated using Molsoft prediction server (http://molsoft.com/mprop/) (31). Canonical SMILES data from PubChem server was used as an input data for Molsoft prediction server, and drug-likeness proprieties were analyzed. 

### Protein active site predictions

Prediction of the active site residues was analyzed by using computed atlas of surface topography of proteins (CASTp) web software (http://cast.engr.uic.edu) (32). CASTp predicts specific amino acid positioning within proteins surface through SwissProt mapping method as well as Online Mendelian Inheritance in Man (OMIM) mapping method (33,34). Finally, we selected some active site residues for further studies: SER630, TYR631, HIS740, ASP708, and TYR547. These preferred residues were used for docking analysis between DPP-4 and the implicated anti-diabetic drugs. 

### Docking analysis and interactions of microenvironment

To understand micro-environment of the interactions between druggable target protein (DPP-4) and anti-diabetic drugs, we performed protein-drug interactions using docking analysis. Molecular docking was carried out by utilizing AutoDock (version 4.2.5.1.) software which uses Lamarckian Genetic Algorithm (LGA) (35). LGA was adopted as a search parameter, derived from adaptive local search. AutoDock-Tools 1.5.6rc3 were used to prepare the protein, ligand, grid parameter file, docking parameter file and to visualize docked structure. Here, free energy during binding has also been analyzed. Thus, it is equal to the variation between i. The energy of ligand and protein in unbound state and ii. The energy of ligand–protein complex. Force field incorporates six pairwise evaluations (V) and was calculated as the conformational entropy lost upon binding (ΔS conf), approximately: 

$$\mathrm{∆G}=\left(V_{\mathrm{bound}}^{\mathrm{L-L}}-V_{\mathrm{unbound}}^{\mathrm{L-L}}\right)+\left(V_{\mathrm{bound}}^{\mathrm{P-P}}-V_{\mathrm{unbound}}^{\mathrm{P-P}}\right)+\left(V_{\mathrm{bound}}^{\mathrm{P-L}}-V_{\mathrm{unbound}}^{\mathrm{P-L}}+{\mathrm{∆S}}_{\mathrm{conf}}\right)$$

In the equation, "ligand" is denoted as L and "protein" is denoted as P during formation of protein-ligand complex. It is also understood that the two molecules are adequately distant from each other and in the unbound situation $V_{\mathrm{unbound}}^{\mathrm{P-L}}$is zero.

The pairwise atomic terms includes evaluations for dispersion/repulsion, H-bonding, electrostatics, and energy. 

$$V=W_{\mathrm{vdw}}\overset{\Sigma }{\mathrm{i,j}}\left(\frac{A_{j}}{r_{j}^{12}}-\frac{B_{j}}{r_{j}^{6}}\right)+W_{\mathrm{hbond}}\overset{\Sigma }{\mathrm{i,j}}\mathrm{E(t)}\left(\frac{C_{j}}{r_{j}^{12}}-\frac{D_{j}}{r_{j}^{10}}\right)+W_{\mathrm{elec}}\overset{\Sigma }{\mathrm{i,j}}\frac{q_{i}q_{j}}{e(r_{j})r_{j}}+W_{\mathrm{sol}}\overset{\Sigma }{\mathrm{i,j}}\left(S_{i}V_{j}+S_{j}V_{i}\right)e^{\left(\frac{{\mathrm{-r}}_{\mathrm{ij}}^{2}}{2\sigma^{2}}\right)}$$

Weighting constants=*W*. It is used to standardize the empirical free energy in a place of experimentally described complexes.

All water molecules and bound ligand N-acetylglucosamine (NAG) have been removed from the original PDB file. H atoms have been added to the protein crystal structure and nonpolar H atoms have been merged. Gasteiger charges were assigned to the ligand and all torsions were accepted to rotate during docking. The grid maps of docking studies were generated using the AutoGrid4 which was implemented in the Autodock4 distribution. Grid center was placed in the middle of the receptor and grid dimensions were 126×88×88 along X, Y, and Z-axis with points separated by 0.442Å. Short range van der Waals’ and electrostatic interactions, H-bonding and entropy losses were included for energy based autodock scoring function. Hundred autonomous docking runs were performed for each ligand molecules. The other parameters for the genetic algorithm (GA) were defined as follows: population size 150; maximum number of 2500000 energy evaluations; mutation; a maximum number of generations of 27,000 and crossover rates of 0.02 and 0.8, correspondingly. Rigid docking was performed for this analysis. We also analyzed micro-environmental factors such as binding energy, intermolecular energy, electrostatic energy, E_VHD_ and LE.

### Analysis of residual interactions at the proteindrug interface

Thereafter, residual interactions at the proteindrug interface with all selected drug models were evaluated using LIGPLOT (v.4.5.3) program, which can plot protein-drug interactions (36,37). Through LIGPLOT, we can demonstrate those interaction points in the plot where H-bonds and hydrophobic contacts intercede. In the plot, H-bonds are shown through the dashed lines between the involved atoms. On the other hand, hydrophobic contacts are symbolized by an arc with spokes radiating in the direction of ligand atoms where they make contact. The contacted atoms are symbolized with spokes radiating back. 

## Results

### Target protein and drugs

**Fig.1:**
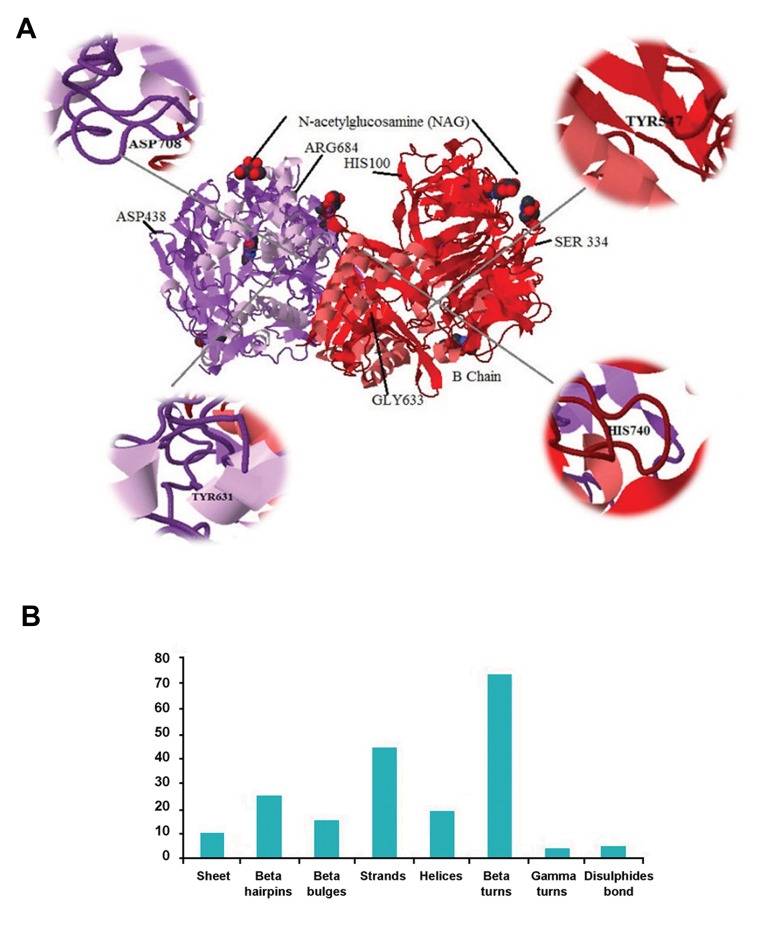
Analysis of target structures in our study. A. The structure of Dipeptidyl Peptidase-IV (DPP-4) with active sites and B. Various secondary structural elements presented in DPP-4.

**Fig.2:**
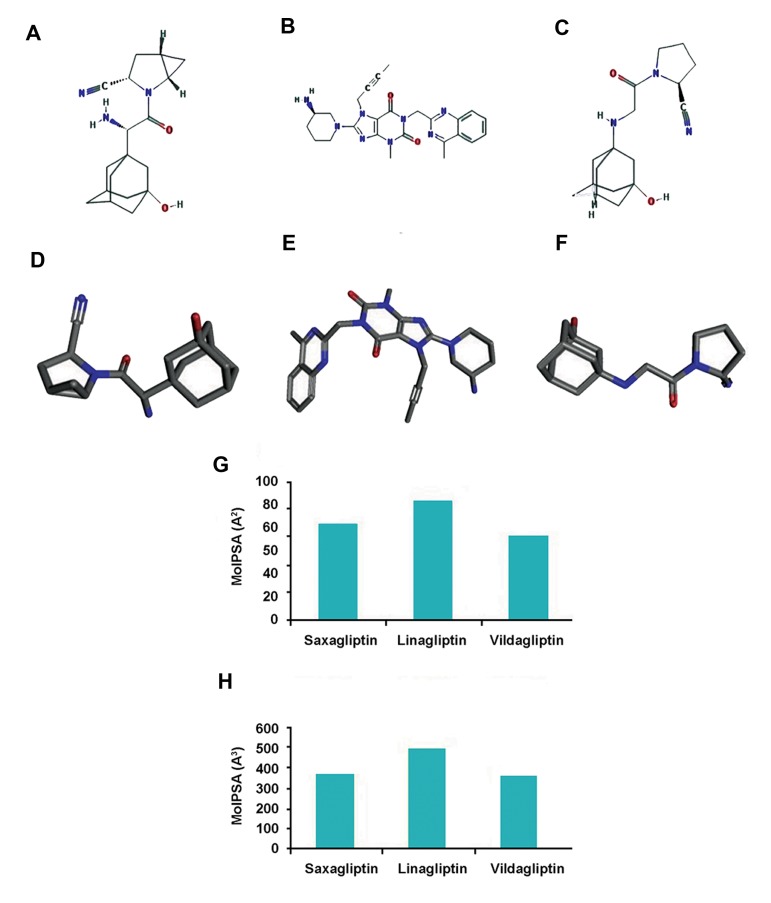
Structure of different anti-diabetic drugs used in our study. A. 2D structure of saxagliptin, B. 2D structure of linagliptin, C. 2D structure of vildagliptin, D. 3D structure of saxagliptin, E. 3D structure of linagliptin, F. 3D structure of vildagliptin, G. Mol PSA (A ^2^) variation for three anti-diabetic drugs showing that linagliptin has highest Mol PSA, and H. MolVol (A ^3^) variation for three anti-diabetic drugs showing that linagliptin has highest MolVol.

Structure of target protein DPP-4 was determined with software Jmol (Fig.1A). The tertiary structural components like alpha-helixes and beta pleatedsheets, beta hairpins, beta bulges, beta turns and disulfide bonds of DPP-4 were also observed (Fig.1B). Meanwhile, 2D structures of saxagliptin, linagliptin and vildagliptin were also generated (Fig.2A-C,respectively). Similarly, 3D structures of saxagliptin, linagliptin and vildagliptin were analyzed and noted in Figure 2D-F, respectively. 

### Drug-likeness analysis 

**Fig.3:**
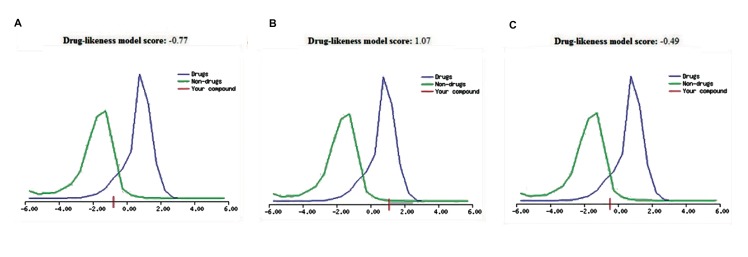
Drug-likeness models of our selected anti-diabetic drugs. A. Drug-likeness models of saxagliptin, B. Drug-likeness models of linaglip- tin, and C. Drug-likeness models of vildagliptin.

Drug-likeness is used to comprehend the drug like properties of a molecule, theoretically. The druglike molecule possesses a logarithm of the partition coefficient called log-P and the value usually lies between the range of 0.4 and 5.6. This property can be utilized to predict the drug like property for any molecule and it is extensively accepted among medicinal chemists (38). In calculation of log-P value for saxagliptin, linagliptin and vildagliptin, we respectively observed a score of 0.77, 1.07 and 0.49 (Fig.3). MolLogP, MolLogS, Mol PSA and MolVol of these three drugs are recorded in the Table 1. Among these drugs, highest log-P value was observed for linagliptin (1.07). 

**Table 1:** Molecular properties of three anti-diabetic drugs


Molecular properties and drug-likeness	Saxagliptin	Linagliptin	Vildagliptin

Molecular formula	C_18_H_25_N_3_O_2_	C_25_H_28_N_8_O_2_	C_17_H_25_N_3_O_2_
Molecular weight (g/mol)	315.19	472.23	303.19
Number of HBA	4	6	4
Number of HBD	3	2	2
MolLogP [in Log(g/mole)]	0.05	1.89	1.28
MolLogS	-3.56 [in Log(moles/L)] 86.44 (in mg/L)	-2.90 [in Log(moles/L)] 591.20 (in mg/L)	-4.38 [in Log(moles/L)] 12.59 (in mg/L)


HBA; Hydrogen bond acceptors and HBD; Hydrogen bond donors.

### Active sites and micro-environment of DPP-4 and anti-diabetic drug interactions

Various possible active site residues on druggable target protein DPP-4 was calculated, which is noted in the supplementary Table 1. We have selected five active site residues for further interaction studies. In our analysis, micro-environmental factors such as binding energy, intermolecular energy, electrostatic energy, E_VHD_ and LE of the drugs and DPP-4 were evaluated.

### Binding energy

**Fig.4:**
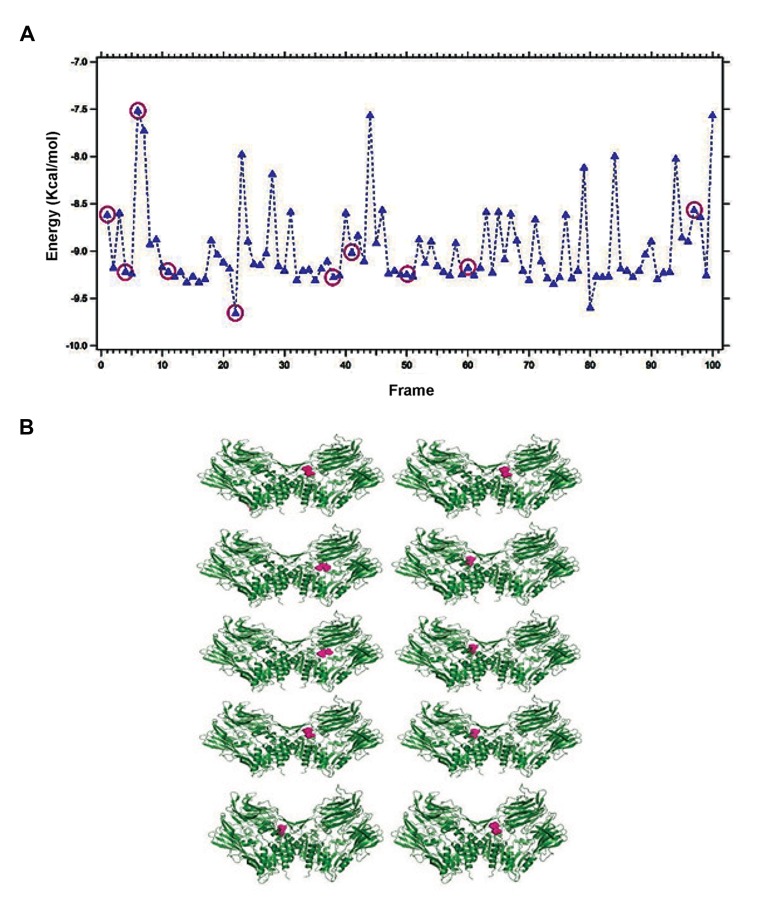
Binding energy during interaction of saxagliptin with Dipeptidyl Peptidase-IV (DPP-4). A. Plotted binding energy for hundred autonomous docking of saxagliptin with DPP-4 and B. Ten chosen conformations from hundred autonomous docking in DPP-4 and saxagliptin interface.

**Fig.5:**
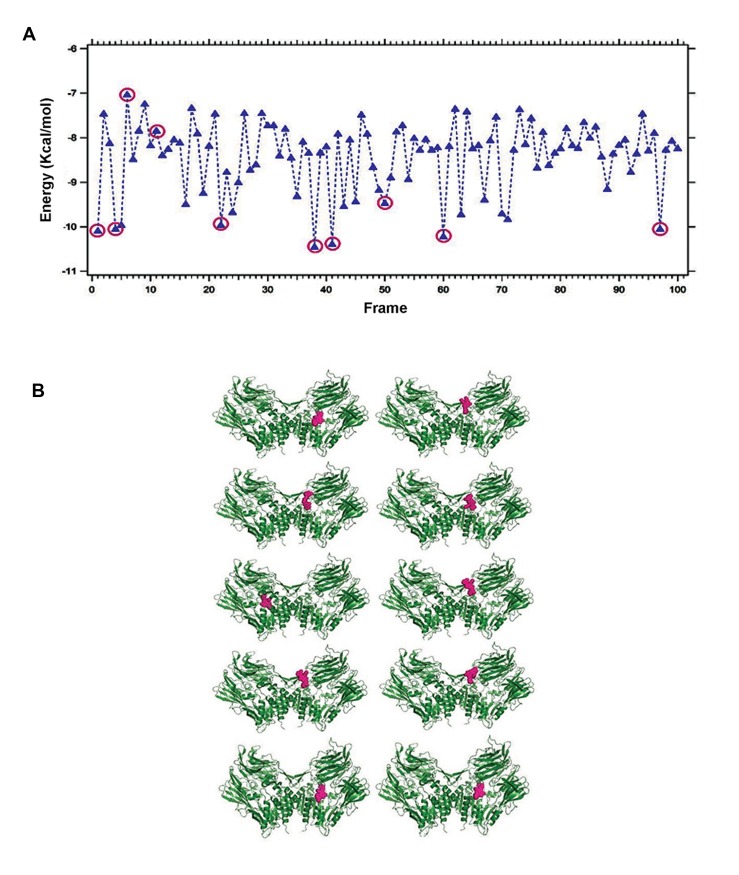
Binding energy during interaction of linagliptin with Dipeptidyl Peptidase-IV (DPP-4). A. Plotted binding energy for hundred au- tonomous docking of linagliptin with DPP-4 and B. Ten chosen conformations from hundred autonomous docking in DPP-4 and linagliptin interface.

**Fig.6:**
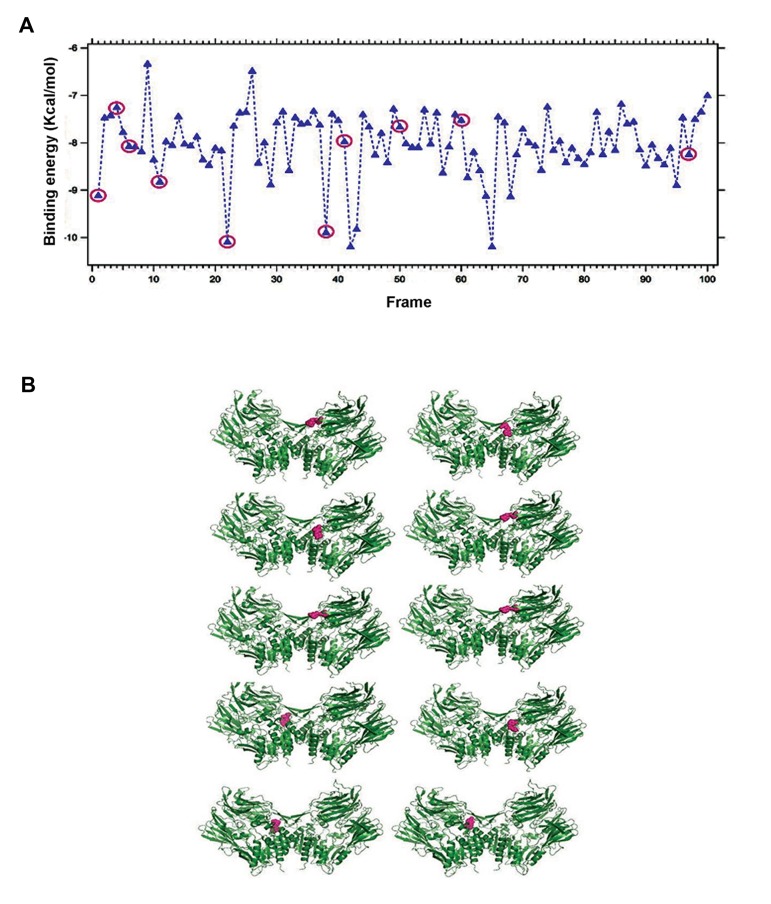
Binding energy during the interaction of vildagliptin with Dipeptidyl Peptidase-IV (DPP-4). A. Plotted binding energy for hundred autonomous docking of vildagliptin with DPP-4 and B. Ten chosen conformations from hundred autonomous docking in DPP-4 and vilda- gliptin interface.

**Fig.7:**
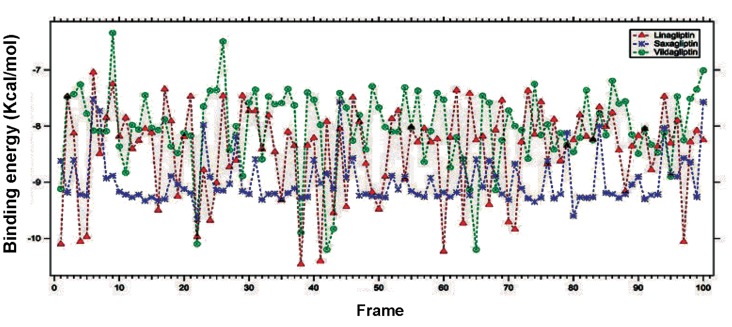
Comparison of three plotted binding energies (saxagliptin, linagliptin and vildagliptin with DPP-4) exposed in a single frame. DPP-4; Dipeptidyl Peptidase-IV (DPP-4).

Binding energy is the energy required for binding of any two molecules and henceforth it is also vital for interactions between any drug and target protein. A little amount of the binding energy is obligatory during drug and target protein interaction, leading to the conformational changes (39). Therefore, binding energy and at least ten binding conformations of DPP4 with saxagliptin (Fig.4A,B), linagliptin (Fig.5A,B) and vildagliptin (Fig.6A,B) from the binding energy frame was analyzed. With the purpose of comparing binding energy between DPP-4 and any of saxagliptin, linagliptin and vildagliptin, we have plotted three binding energies (Figs.4A, 5A, 6A) altogether in a single frame as shown in the Figure 7. Upon comparing, lowest binding energy was determined for linagliptin with DPP-4 binding plot, after hundreds of docking runs. On the other hand, the overall binding energy of saxagliptin appears to be low with DPP-4 as evidenced from plot after hundreds of docking runs. 

### Intermolecular energy

**Fig.8:**
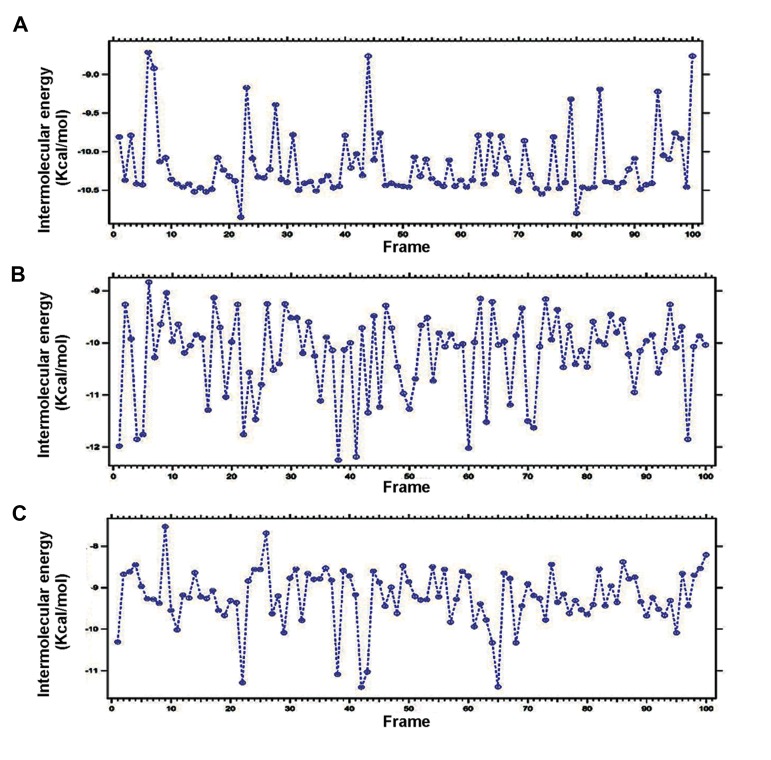
Plotted intermolecular energy for three anti-diabetic drugs during interaction with Dipeptidyl Peptidase-IV (DPP-4). A. Plotted in- termolecular energy for saxagliptin during interaction with DPP-4, B. Plotted intermolecular energy for linagliptin during interaction with DPP-4, and C. Plotted intermolecular energy for vildagliptin during interaction with DPP-4.

Intermolecular energy denotes type of interactions between drugs and residues in a target, while no atomic bond is formed during these interactions. A drug when binds into a protein binding site contains intermolecular translation, rotation and intramolecular conformational changes (40). The intermolecular energy during the interaction of DPP-4 with saxagliptin, linagliptin and vildagliptin is recorded in the Figure 8A-C, respectively. 

### Electrostatic energy

**Fig.9:**
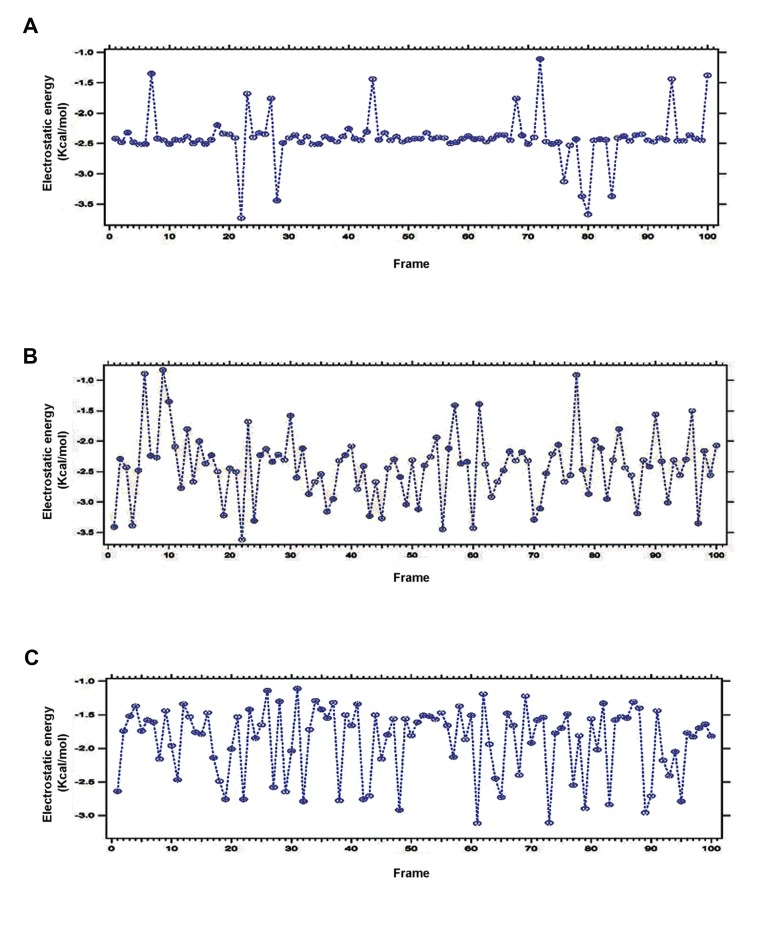
Plotted electrostatic energy for three anti-diabetic drugs during interaction with Dipeptidyl Peptidase-IV (DPP-4). A. Electrostatic energy plot for saxagliptin during interaction with DPP-4, B. Intermolecular electrostatic plot for linagliptin during interaction with DPP-4, and C. Plotted electrostatic energy for vildagliptin during interaction with DPP-4.

Electrostatic interactions are one of the important factors for drug target as well as for drug binding. For computer-based understanding of protein energies, electrostatic energy is one of the crucial factors which should be considered to understand the biological function of a molecule (21). Therefore, electrostatic energy during interaction of DPP-4 with saxagliptin, linagliptin and vildagliptin was analyzed and are recorded in the Figure 9A-C, respectively. 

### Van der Waals’+H-bond+desolvo energy

**Fig.10:**
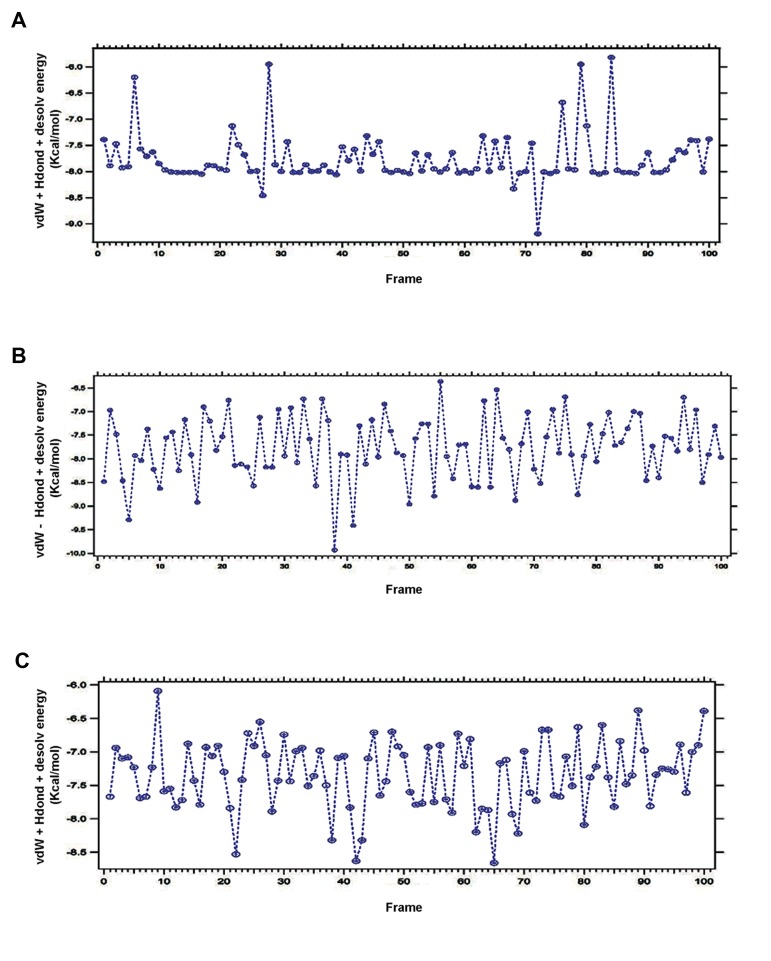
Plotted E_VHD_ for anti-diabetic drugs. A. Plotted E_VHD_ for saxagliptin, B. Plotted E_VHD_ for linagliptin, and C. Plotted E_VHD_ for vildagliptin.

Van der Waals interactions are perhaps the most basic type of interactions that exists between any two molecules (41). Free energy changes (ΔG) are an amalgamation of changes in enthalpy (ΔH) and entropy (ΔS) together, and both enthalpy and entropy must be measured during the time of binding (42). Through interaction, E_VHD_ of DPP-4 with saxagliptin, linagliptin and vildagliptin was observed (Fig.10A-C, respectively).

### Ligand efficiency

LE metrics help to understand the molecular properties which are required to calculate binding affinity for a drug target. Thus, it has a great role in improving the quality of drug during current drug discovery practices (43). LE metrics can be defined mathematically, as follow: 

LE=(ΔG)/N

Actually LE is the ratio of Gibbs free energy (ΔG) to the number of non-hydrogen atoms of the compound (N), while we consider that ΔG=-RTlnKi (44). The equation can be altered as follow (45): 

$$\mathrm{LE}=1.4({\mathrm{-logIC}}_{50})/N$$

**Fig.11:**
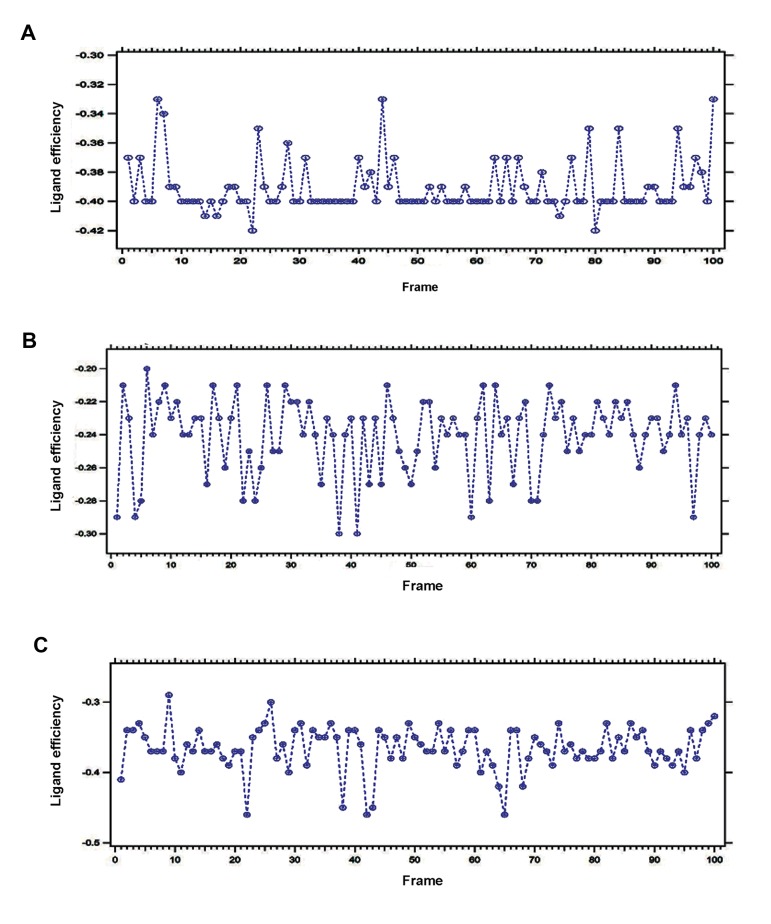
Plotted ligand efficiency for three anti-diabetic drugs during interaction with Dipeptidyl Peptidase-IV (DPP-4). A. Plotted ligand efficiency for saxagliptin, B. Plotted ligand efficiency for linagliptin, and C. Plotted ligand efficiency for vildagliptin.

During the interaction, LE of DPP-4 with saxagliptin, linagliptin and vildagliptin was measured and illustrated in the Figure 11A-C, respectively.

### Analysis of residual interactions at the proteindrug interface

**Fig.12:**
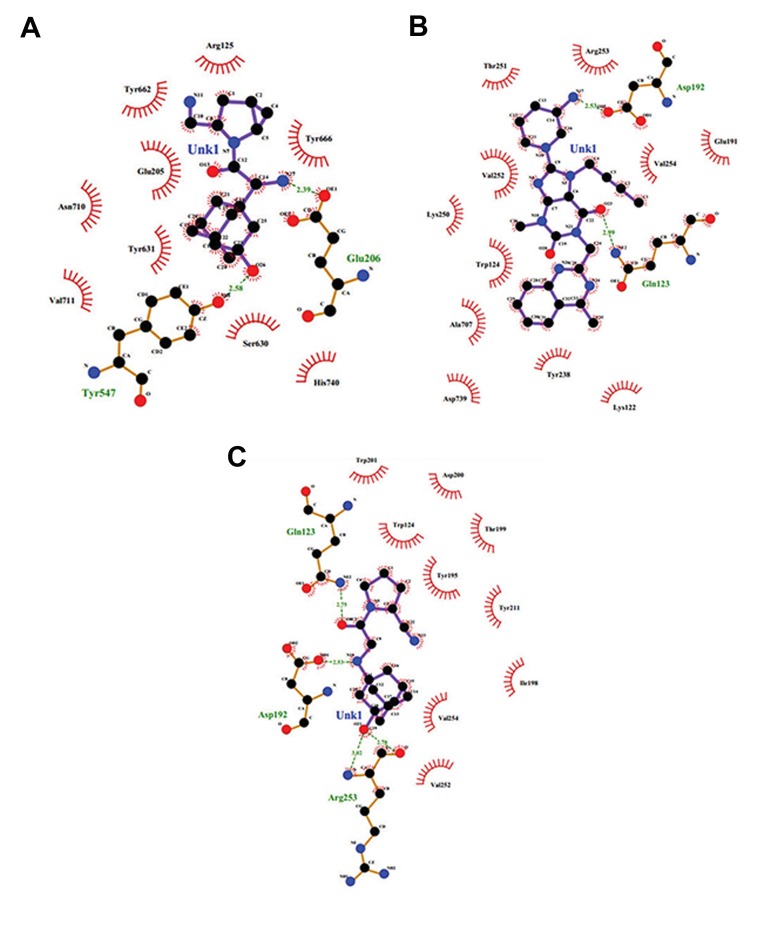
Residual interactions at Dipeptidyl Peptidase-IV (DPP-4) protein-anti-diabetic drug interface. A. Residual interactions at the DPP- 4-saxagliptin interface, B. Residual interactions at the DPP-4–linagliptin interface, and C. Residual interactions at the DPP-4–vildagliptin interface.

The binding site of ligands, saxagliptin, linagliptin and vildagliptin, are shown in the Figure 12A-C, respectively. LIGPLOT (protein ligand) diagrams showed interaction between drugs and active site residues of protein using H-bonding and hydrophobic contacts. Here, we have calculated the numbers of H-bonds that are formed between the active site of DPP4 and drugs. These are two, in terms of the number, in the case of DPP-4 and saxagliptin interaction (Fig.12A); two in the case of DPP4 and linagliptin interaction (Fig.12B) and four in the case of DPP4 and vildagliptin interaction (Fig.12C). Some residues are also involved in few hydrophobic interactions in all cases. 

### Discussion

In the middle of 1990s, inactivation of GLP-1 receptor through DPP-4 was recognized. After invention, GLP-1-based treatment for T2D had been paid substantive attention (25, 46). Using DPP-4 as drug target, several anti-diabetic drugs were developed especially, sitagliptin (brand name: Januvia), saxagliptin (brand name: Onglyza), linagliptin (brand name: Tradjenta) and alogliptin (brand name: Nesina). All of these drugs are already available and administered in clinical practices (47). In this study, we used three drugs (i.e. saxagliptin, linagliptin and vildagliptin) to understand the micro-environmental signature of the interactions with target DPP-4.

Researchers from two pharmaceutical companies (i.e. Pfizer and Vertex) described the character and structural appearance of any molecule which may render them more or less drug like properties (48, 49). During process of drug discovery, the objective of drug-likeness analysis is to apply absorption, distribution, metabolism and excretion (ADME). Drug-likeness analysis is a crucial step before pre-clinical development to avoid costly experiments and to make the process more cost effective (50).

The "rule-of-five" indicated that most of the orally administered drugs have to possess the following five properties: i. Molecular weight (MW) should be 500 g/mol or less, ii. log-P value should not be higher than five, iii. Presence of five or fewer H-bond donor sites, iv. Presence of 10 or less H-bonds in the acceptor sites (in case Nitrogen and Oxygen atoms), and v. Poor permeability, if any of the mentioned factors exceeds the indicated limits (50). Moreover, it is also very difficult for central nervous system applicable compounds to cross over blood brain barrier and reach the target site. In general, drug like properties can at least predict a compound to possess basic drug like characteristics and thus it can make the process more economical. Here, in this study all three tested drugs demonstrated a score lying well within the range of drug-like properties. Data of drug-likeness models revealed that linagliptin has the highest drug like score and properties among all tested drugs.

Binding a drug with its target protein sites is the event which directly relates to the medicinal activity it possess. Anti-diabetic drugs (saxagliptin, linagliptin and vildagliptin) bind with target protein DPP-4 leading to anti-diabetic activity. Currently, it is a major challenge for drug designing process that drugs or drug like compounds selectively binds to their proper target, while it should not cause any side-effect by binding to the other similar receptors (13, 51). The amount of anti-diabetic drug and target protein, DPP-4, as well as the ultimate complex formed by them determines the anti-diabetic activity displayed by that compound. The formed complex can be described by the equilibrium binding expression as shown below:

Anti-diabetic drug+DPP-4 ⇌ Anti-diabetic drug.DPP-4

$$\mathrm{Keq}=\frac{\mathrm{[Anti-diabetic drug. DPP-4]}}{\mathrm{[Anti-diabetic drug]}\mathrm{[DPP-4]}}$$

The value Keq is identical to Ka (association constant) and Kb (binding constant), and therefore:

$$K_{\mathrm{eq}}=K_{a}=K_{b}$$

Binding of drugs with their targets in the cell depend on the interactions formed by micro- environmental factors, such as the formation of energetically favorable bonding interactions between two partners. The equation for the free energy of binding (ΔG) to Keq is shown below:

ΔG =*-RTInK_eq_*

Here, R is the gas constant (1.987 calK- 1mol-1) and T is the absolute temperature, which is generally considered as room temperature, 298.15˚K (52). In this equation, the inhibition constant is denoted for the dissociation reaction which is EI E+I , whereas DGobs refers as the reverse process of binding, E+I EI; where E is the enzyme and I is the inhibitor. The free energy of binding (ΔG) is equal to:

ΔG =*-RTInK_eq_*

ΔH is the enthalpy of binding and represents the energetic gains of existing bonds. S is the entropy of binding and is used to calculate disorder in the system (18). Previously, Metzler et al. (53) described the inhibition of DPP-4 by saxagliptin through formation of a histidine-assisted covalent bond but reversible complex. Here, we have described the interaction energies between ligand drugs (saxagliptin, linagliptin and vildagliptin)- receptor (DPP-4) and have analyzed a free energy- based expression. During these interactions, we observed that binding energy for DPP-4 and linagliptin was lowest among these three ligands. Therefore, linagliptin and DPP-4 is proposed to form a more stable interaction.

Furthermore, we recorded the number of involved residues during drug-protein interaction. We observed that the residues like Arg125, Glu205, Trr661, Ser630, Tyr631, Tyr666, Asn710, Val711 and His740 were involved in hydrophobic interactions for DPP-4 and saxagliptin. Similarly, the residues involved in hydrophobic interactions for DPP-4 and linagliptin were Glu191, Lys122, Trp124, Tyr238, Lys250, Thr251, Val252, Arg253, Val254, Ala707, and Asp739. Involved residues in hydrophobic interactions of DPP-4 and vildagliptin were Trp124, Trp195, IIe198, Thr199, Asp200, Trp201, Trp211, Val252, and Val254. Here, we found that more residues were involved in hydrophobic interactions than H-bond formation for the studied protein–drug interaction. This result corroborates with our previous observations (25).

## Conclusion

Druggability of a target protein is its ability to be modulated as drug-like molecule. Here, we have tried to understand the druggability of DPP- 4 to depict micro-environmental signature of the interactions between target protein and drugs. DPP- 4 is a confirmed and validated target for treatment of T2D which has received significant interest from the pharmaceutical companies over the last few years. Our in silico analysis for drug-target interaction demonstrated that linagliptin possess lowest binding energy with DPP-4 among the tested anti-diabetic drugs (saxagliptin, linagliptin and vildagliptin). Taken together, linagliptin appears to be the best available drug among three anti-diabetic drugs with reference to the drug-target interactions and micro-environmental signature point of view. 

We hope that diabetic patients will be benefited soon once we understand a stable interaction between an anti-diabetic drug and target. Other than that, our model is important for the target selection phase as well as small molecule selection phase. This effective inhibitor micro- environmental signature model of DPP-4 may yield several new compounds toward discovery of new anti-diabetic drugs.

## References

[1] Bunnage ME (2011). Getting pharmaceutical R&D back on target. Nat Chem Biol.

[2] Lessl M, Bryans JS, Richards D, Asadullah K (2011). Crowd sourcing in drug discovery. Nat Rev Drug Discov.

[3] Lessl M, Schoepe S, Sommer A, Schneider M, Asadullah K (2011). Grants4Targets an innovative approach to translate ideas from basic research into novel drugs. Drug Discov Today.

[4] Liu T, Altman RB (2014). Identifying druggable targets by protein microenvironments matching: application to transcription factors. CPT Pharmacometrics Syst Pharmacol.

[5] Drews J (1996). Genomic sciences and the medicine of tomorrow. Nat Biotechnol.

[6] Hopkins AL, Groom CR (2002). The druggable genome. Nat Rev Drug Discov.

[7] Golden JB (2003). Prioritizing the human genome: knowledge management for drug discovery. Curr Opin Drug Discov Devel.

[8] Golden J (2003). Towards a tractable genome: knowledge management in drug discovery. Curr Drug Discov.

[9] Imming P, Sinning C, Meyer A (2006). Drugs, their targets and the nature and number of drug targets. Nat Rev Drug Discov.

[10] Zheng C, Han L, Yap CW, Xie B, Chen Y (2006). Progress and problems in the exploration of therapeutic targets. Drug Discov Today.

[11] Zheng CJ, Han LY, Yap CW, Ji ZL, Cao ZW, Chen YZ (2006). Therapeutic targets: progress of their exploration and investigation of their characteristics. Pharmacol Rev.

[12] Overington JP, Al-Lazikani B, Hopkins AL (2006). How many drug targets are there?. Nat Rev Drug Discov.

[13] Lipinski CA, Lombardo F, Dominy BW, Feeney PJ (2001). Experimental and computational approaches to estimate solubility and permeability in drug discovery and development settings. Adv Drug Deliv Rev.

[14] Sakharkar MK, Sakharkar KR (2007). Targetability of human disease genes. Curr Drug Discov Technol.

[15] Sakharkar MK, Sakharkar KR, Pervaiz S (2007). Druggability of human disease genes. Int J Biochem Cell Biol.

[16] Arrowsmith J (2011). Trial watch: phase II failures: 2008-2010. Nat Rev Drug Discov.

[17] Datta S, Halder M (2014). Detailed scrutiny of the anion receptor pocket in subdomain IIA of serum proteins toward individual response to specific ligands: HSA-pocket resembles flexible biological slide-wrench unlike BSA. J Phys Chem B.

[18] Henrich S, Feierberg I, Wang T, Blomberg N, Wade RC (2010). Comparative binding energy analysis for binding affinity and target selectivity prediction. Proteins.

[19] Leckband D (2000). Measuring the forces that control protein interactions. Annu Rev Biophys Biomol Struct.

[20] Eckenhoff RG, Johansson JS (1997). Molecular interactions between inhaled anesthetics and proteins. Pharmacol Rev.

[21] Kukic P, Nielsen JE (2010). Electrostatics in proteins and proteinligand complexes. Future Med Chem.

[22] Panigrahi SK (2008). Strong and weak hydrogen bonds in protein-ligand complexes of kinases: a comparative study. Amino Acids.

[23] Abad-Zapatero C (2007). Ligand efficiency indices for effective drug discovery. Expert Opin Drug Discov.

[24] Chakraborty C, Roy SS, Hsu MJ, Agoramoorthy G (2011). Landscape mapping of functional proteins in insulin signal transduction and insulin resistance: a network-based protein-protein interaction analysis. PLoS One.

[25] Chakraborty C, Hsu MJ, Agoramoorthy G (2014). Understanding the molecular dynamics of type-2 diabetes drug target DPP-4 and its interaction with Sitagliptin and inhibitor Diprotin-A. Cell Biochem Biophys.

[26] Whiting DR, Guariguata L, Weil C, Shaw J (2011). IDF diabetes atlas: global estimates of the prevalence of diabetes for 2011 and 2030. Diabetes Res Clin Pract.

[27] Wild S, Roglic G, Green A, Sicree R, King H (2004). Global prevalence of diabetes: estimates for the year 2000 and projections for 2030. Diabetes Care.

[28] Scheen AJ (2012). A review of gliptins in 2011. Expert Opin Pharmacother.

[29] Berman HM, Kleywegt GJ, Nakamura H, Markley JL (2014). The protein data bank archive as an open data resource. J Comput Aided Mol Des.

[30] Knox C, Law V, Jewison T, Liu P, Ly S, Frolkis A (2011). DrugBank 3.0: a comprehensive resource for 'omics’ research on drugs. Nucleic Acids Res.

[31] Arnautova YA, Abagyan RA, Totrov M (2011). Development os a new physics-based internal coordinate mechanics force field and its application to protein loop modeling. Proteins: Struct Funct Bioinf.

[32] Dundas J, Ouyang Z, Tseng J, Binkowski A, Turpaz Y, Liang J (2006). CASTp: computed atlas of surface topography of proteins with structural and topographical mapping of functionally annotated residues. Nucleic Acids Res.

[33] Porter CT, Bartlett GJ, Thornton JM (2004). The Catalytic Site Atlas: a resource of catalytic sites and residues identified in enzymes using structural data. Nucleic Acids Res.

[34] Furnham N, Holliday GL, de Beer TA, Jacobsen JO, Pearson WR, Thornton JM (2014). The Catalytic Site Atlas 2.0: cataloging catalytic sites and residues identified in enzymes. Nucleic Acids Res.

[35] Fuhrmann J, Rurainski A, Lenhof HP, Neumann D (2010). A new Lamarckian genetic algorithm for flexible ligand-receptor docking. J Comput Chem.

[36] Wallace AC, Laskowski RA, Thornton JM (1995). LIGPLOT: a program to generate schematic diagrams of protein-ligand interactions. Protein Eng.

[37] Laskowski RA, Swindells MB (2011). LigPlot+: multiple ligandprotein interaction diagrams for drug discovery. J Chem Inf Model.

[38] Lipinski CA (2004). Leadand drug-like compounds: the rule-of-five revolution. Drug Discov Today Technol.

[39] Teague SJ (2003). Implications of protein flexibility for drug discovery. Nat Rev Drug Discov.

[40] Leach AR, Shoichet BK, Peishoff CE (2006). Prediction of protein-ligand interactions.Docking and scoring: successes and gaps. J Med Chem.

[41] Van Oss CJ, Good RJ, Chaudhury MK (1986). The role of van der Waals forces and hydrogen bonds in “hydrophobic interactions” between biopolymers and low energy surfaces. J Colloid Interface Sci.

[42] López-Lucendo MF, Solís D, André S, Hirabayashi J, Kasai K, Kaltner H (2004). Growth-regulatory human galectin1:crystallographic characterisation of the structural changes induced by single-site mutations and their impact on the thermodynamics of ligand binding. J Mol Biol.

[43] Hopkins AL, Keseru GM, Leeson PD, Rees DC, Reynolds CH (2014). The role of ligand efficiency metrics in drug discovery. Nat Rev Drug Discov.

[44] Hopkins AL, Groom CR, Alex A (2004). Ligand efficiency: a useful metric for lead selection. Drug Discovery Today.

[45] Shultz MD (2013). Setting expectations in molecular optimizations: strengths and limitations of commonly used composite parameters. Bioorg Med Chem Lett.

[46] Albrechtsen NJW, Kuhre RE, Deacon CF, Holst JJ (2014). Targeting the intestinal L-cell for obesity and type 2 diabetes treatment. Expert Rev Endocrinol Metab.

[47] Bohannon N (2009). Overview of the gliptin class (dipeptidyl peptidase-4 inhibitors) in clinical practice. Postgrad Med.

[48] Jorgensen WL (2004). The many roles of computation in drug discovery. Science.

[49] Walters WP, Murcko MA (2002). Prediction of 'drug-likeness’. Adv Drug Deliv Rev.

[50] Egan WJ, Merz KM Jr, Baldwin JJ (2000). Prediction of drug absorption using multivariate statistics. J Med Chem.

[51] Lipinski CA, Lombardo F, Dominy BW, Feeney PJ (2001). Experimental and computational approaches to estimate solubility and permeability in drug discovery and development settings. Adv Drug Deliv Rev.

[52] Atkins PW (1982). Physical chemistry.

[53] Metzler WJ, Yanchunas J, Weigelt C, Kish K, Klei HE, Xie D (2008). Involvement of DPP-IV catalytic residues in enzyme-saxagliptin complex formation. Protein Sci.

